# Hematological Complications of Human Immunodeficiency Virus (HIV) Infection: An Update From an HIV-Endemic Setting

**DOI:** 10.1093/ofid/ofae162

**Published:** 2024-03-19

**Authors:** Jessica Opie, Estelle Verburgh, Jenique Bailly, Elizabeth Mayne, Vernon Louw

**Affiliations:** Division of Haematology, Department of Pathology, University of Cape Town, Cape Town, South Africa; National Health Laboratory Service, Groote Schuur Hospital, Cape Town, South Africa; Division of Clinical Haematology, Department of Medicine, University of Cape Town, Cape Town, South Africa; Division of Clinical Haematology, Department of Medicine, Groote Schuur Hospital, Cape Town, South Africa; Division of Haematology, Department of Pathology, University of Cape Town, Cape Town, South Africa; National Health Laboratory Service, Groote Schuur Hospital, Cape Town, South Africa; National Health Laboratory Service, Groote Schuur Hospital, Cape Town, South Africa; Division of Immunology, Department of Pathology, University of Cape Town, Cape Town, South Africa; Division of Clinical Haematology, Department of Medicine, University of Cape Town, Cape Town, South Africa; Division of Clinical Haematology, Department of Medicine, Groote Schuur Hospital, Cape Town, South Africa

**Keywords:** aggressive lymphoma, bone marrow, cytopenias, drug effects, HIV

## Abstract

Medical professionals, particularly in regions with a high burden of human immunodeficiency virus (HIV), should be alert to the hematological complications of HIV, which may include cytopenias, malignancy, and coagulation disturbances. Patients may present with these conditions as the first manifestation of HIV infection. Hematological abnormalities are often multifactorial with opportunistic infections, drugs, malignancy, and HIV infection itself contributing to the clinical presentation, and the diagnosis should consider all these factors. Life-threatening hematological complications requiring urgent diagnosis and management include thrombotic thrombocytopenic purpura, superior mediastinal syndrome, spinal cord compression, and tumor lysis syndrome due to aggressive lymphoma. Antiretroviral therapy is the therapeutic backbone, including for patients with advanced HIV, in addition to specific therapy for the complication. This article reviews the impact of HIV on the hematological system and provides a clinical and diagnostic approach, including the role of a bone marrow biopsy, focusing on perspectives from sub-Saharan Africa.

## Teaser

Summary: Cytopenias, often multifactorial, are common in HIV. HIV markedly increases the incidence of aggressive lymphoma. A bone marrow biopsy is a useful diagnostic tool for unexplained cytopenias. HIV increases risks for thrombotic thrombocytopenic purpura, cardiovascular events, and venous thromboembolism.

Approximately 39 million people globally were living with human immunodeficiency virus (HIV) in 2022. Sub-Saharan Africa accounts for more than half the global cases [1]. HIV testing coverage has been expanded successfully in sub-Saharan Africa, although only approximately 75% of people with HIV (PWH) receive antiretroviral therapy (ART) [2]. Initiation of ART, even for PWH with advanced disease, markedly improves prognosis and survival and reduces HIV transmission, but HIV-related comorbidities remain a challenge [3].

Hematological abnormalities are common and diverse in PWH, with cytopenias (anemia, leukopenia, thrombocytopenia, or a combination) being the most frequent [4]. Cytopenias are typically multifactorial in etiology [5]. PWH are predisposed to opportunistic infections and hematological malignancies, with a markedly increased risk of both aggressive B-cell non-Hodgkin lymphoma (NHL) and Hodgkin lymphoma (HL), which persists in the ART era [6]. South Africa (SA) has a high burden of HIV-associated tuberculosis (TB), and according to the World Health Organization, more than half of South Africans diagnosed with TB in 2021 were coinfected with HIV [7]. TB has overlapping clinical features with aggressive B-cell lymphomas, including weight loss, night sweats, and lymphadenopathy, which may delay diagnosis and appropriate treatment of lymphoma, since patients are frequently treated with empiric TB therapy in resource-constrained settings [8].

Coagulation is dysregulated in HIV, and PWH have prothrombotic abnormalities due to chronic inflammation and endothelial cell dysfunction [9]. People with HIV therefore have increased rates of cardiovascular disease and venous thromboembolism (VTE) [9, 10]. Severe thrombocytopenia may occur in the setting of HIV-associated immune thrombocytopenia or thrombotic thrombocytopenic purpura (TTP).

This review provides a summary of the common complications of HIV, their underlying mechanisms, and a clinical approach, focusing on perspectives from sub-Saharan Africa.

##  

### SEARCH STRATEGY AND SELECTION CRITERIA

References for this review were identified through searches of PubMed for articles published in English with the search terms “HIV” and “hematopoiesis,” “hematological complications,” “cytopenias,” “anemia,” “thrombocytopenia,” “immune thrombocytopenia,” “leucopenia,” “hemolysis,” “thrombosis,” “thrombotic thrombocytopenic purpura,” “antiretroviral therapy,” “opportunistic infection,” “tuberculosis,” “malignancies,” “cancer,” “B-cell lymphoma,” “Hodgkin lymphoma,” “bone marrow,” “plasmablastic,” “myeloma,” “coagulation,” and “cardiovascular disease” from 2003 to November 2023. Only English-language publications were reviewed.

## CYTOPENIAS

**Figure 1.**
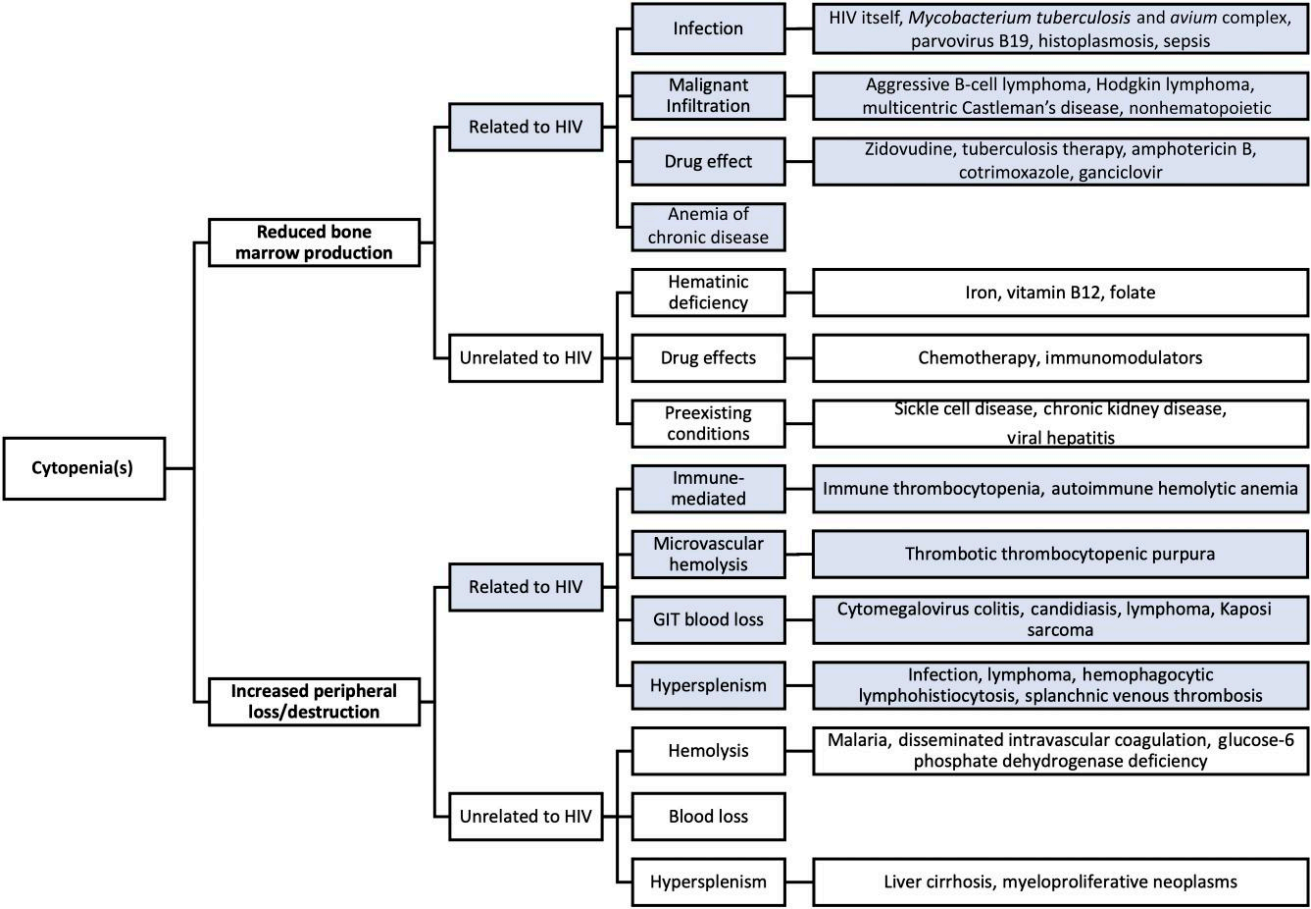
Differential diagnosis of cytopenias in people with human immunodeficiency virus. Abbreviations: GIT, gastrointestinal; HIV, human immunodeficiency virus.

The prevalence of cytopenias, particularly anemia, increases in advanced stages of HIV with lower CD4^+^ T-cell counts, and is ameliorated by ART [4, 11]. Anemia is the most common cytopenia, with a median prevalence of 30% in ART-naive PWH in sub-Saharan Africa [5]. The prevalence of thrombocytopenia and neutropenia in PWH in the SA setting is reported at 12.1% and 21.6%, respectively [12]. Advanced HIV/AIDS, defined as a CD4^+^ T-cell count <200 cells/µL, is a common cause of pancytopenia even in the absence of coexisting pathology [13]. Morphological dysplasia of 1 or more cell lines seen on the blood smear and bone marrow (BM) is a frequent finding in advanced HIV disease and is associated with cytopenias [14]. An approach to the differential diagnosis of cytopenias in PWH is provided in Figure 1.

### Anemia

Common causes of anemia in sub-Saharan Africa include hematinic deficiencies, malaria, chronic kidney disease, and hemoglobinopathies (such as sickle cell disease). Anemia is often a presenting complication in PWH and may be broadly approached as due to decreased BM production and/or increased peripheral red cell destruction/loss (Figure 1).

ART reduces the prevalence of anemia with a reduction in prevalence approaching 45% after 1 year of therapy [15]. HIV causes apoptosis of hematopoietic precursors by infecting hematopoietic stem cells. Furthermore, HIV leads to hematopoietic dysregulation by altering secretion of critical cytokines and growth factors [16–18]. This dysregulation, specifically associated with elevated interleukin 6 and hepcidin levels, reduces bioavailability of iron, resulting in anemia of chronic disease. Anemia of chronic disease is more common with advanced HIV disease and other comorbid conditions, especially coinfection with TB [19]. ART partially reverses cytokine disturbances, leading to hematopoietic recovery and improved peripheral blood counts [20]. Parvovirus B19 infection is an opportunistic infection that directly infects red cell precursors, leading to pure red cell aplasia (PRCA) and severe anemia. In PRCA, erythroid activity is severely reduced with an associated severe reticulocytopenia [21]. Zidovudine, lamivudine, and emtricitabine may rarely cause reversible PRCA with severe anemia, mimicking parvoviral infection [22–24].

HIV may also precipitate autoimmune hemolytic anemia (AIHA), which is caused by autoantibodies binding to autologous red cell antigens, resulting in premature splenic red cell destruction [25]. AIHA is characterized by spherocytic red cells on the blood smear and a positive direct antiglobulin (Coombs) test. Nonspecific biochemical markers of hemolysis are characteristic, such as raised lactate dehydrogenase (LDH) and reduced haptoglobin levels. HIV-associated AIHA usually responds to ART and corticosteroid therapy [26].

BM infiltration, by opportunistic infection and/or malignancy, may displace normal hematopoiesis, leading to anemia and other cytopenias (Figure 1). The commonest coinfection in the BM in PWH is *Mycobacterium tuberculosis* (TB), with *Mycobacterium avium* complex and fungal infection including *Cryptococcus neoformans* and *Histoplasma capsulatum* occurring more rarely [27, 28]. These infections typically cause reactive granuloma formation. High-grade B-cell NHL and HL may also infiltrate the BM in PWH. HL specifically is associated with frequent BM involvement in PWH and more severe anemia than in HL patients without HIV [29, 30]. In a proportion of cases, TB and lymphoma are simultaneously present at diagnosis [29]. Other rarer infiltrative processes include conditions like multicentric Castleman disease, a lymphoproliferative disorder seen in PWH coinfected with human herpesvirus 8 (HHV8), which commonly presents with lymphadenopathy, splenomegaly, B symptoms, and cytopenias [31].

**Figure 2.**
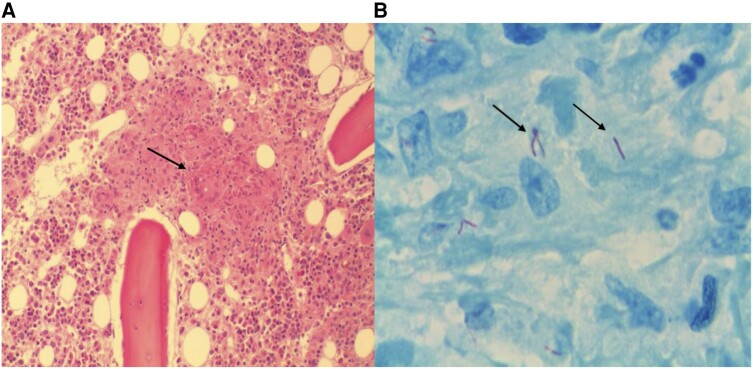
Bone marrow trephine biopsy findings in a person with human immunodeficiency virus presenting with pancytopenia and fever. *A*, Hematoxylin and eosin stain shows an ill-formed granuloma containing a multinucleate giant cell (arrow). *B*, Ziehl-Neelsen stain shows scanty acid-fast bacilli (arrows), characteristic for *Mycobacterium tuberculosis*.

In suspected BM infiltration, a BM biopsy provides a morphological diagnosis with a rapid turnaround time. There is a high diagnostic yield for BM biopsies in PWH, especially in advanced HIV disease presenting with unexplained cytopenias and fever, with disseminated mycobacterial infection being the commonest diagnosis (Figure 2) [27]. Mycobacterial and fungal cultures of the BM aspirate are essential in PWH with advanced HIV, cytopenias, and/or fevers but have a longer turnaround time than morphology [27].

### Leukopenia

The normal absolute neutrophil count is lower in black Africans although reference intervals in sub-Saharan Africa are usually adopted from European populations [32]. In some parts of West Africa, approximately 70% of individuals are Duffy null or Fy (a^–^b^–^), which is protective against malaria but is associated with neutropenia. Duffy null–associated neutropenia, defined as a neutrophil count <1.5 × 10^9^/L, is present in 10%–15% of Duffy-null individuals and in a proportion the neutropenia is severe (<0.5 × 10^9^/L) [33]. When interpreting neutrophil counts in sub-Saharan Africa, these factors should be considered [32, 34].

Neutropenia is a frequent finding in PWH, although an isolated neutropenia (without other cytopenias) is rare. In the Women's Interagency HIV Study, 44% of women had a neutrophil count <2.0 × 10^9^/L at baseline, although this was <1.0 × 10^9^/L in only 7% of patients. During follow-up, neutrophil counts of <2.0 × 10^9^/L and <1.0 × 10^9^/L were seen in 79% and 31% of cases, respectively [35]. Factors associated with neutropenia included advanced HIV disease with CD4^+^ T cell counts <200 cells/µL and HIV viral loads >100 000 copies/mL. Neutropenia was not, however, associated with reduced survival in multivariate analysis [35]. A severe neutropenia of <0.5 × 10^9^/L may be seen in advanced HIV/AIDS, with opportunistic infection or malignancy and/or associated with drugs including ART, antimicrobial agents, and chemotherapy [36, 37] (Figure 1 and Table 1). These causes should be considered and appropriately managed in the first instance. In cases where the cause is not apparent or the neutropenia does not respond to changes in drug management, a BM biopsy may be of value. A neutrophil count <0.25 × 10^9^/L is considered an acceptable threshold for commencing granulocyte colony-stimulating factor therapy [38, 39]. An HIV-associated neutropenic fever with neutrophils <0.5 × 10^9^/L requires urgent empiric broad-spectrum antibiotic therapy [35].

**Table 1.** Common or Severe Drug-Induced Cytopenias in People With Human Immunodeficiency Virus

Drug Class	Drug Name	Indication	Cytopenia(s)
Nucleoside/nucleotide reverse transcriptase inhibitor	Lamivudine, emtricitabine	Part of first-line ART regimen	Anemia (PRCA^a^)
	Zidovudine	Part of first-line ART regimen if tenofovir disoproxil fumarate and abacavir unavailable or contraindicated	Anemia (PRCA^a^), neutropenia Avoid if Hb <8 g/dL, neutrophils <0.75 × 10^9^/L
Antiviral	Ganciclovir, valganciclovir	Cytomegalovirus infection	Neutropenia, thrombocytopenia, pancytopenia
Sulphonamide	Cotrimoxazole (Bactrim)	Pneumocystis, toxoplasmosis prophylaxis/treatment	Megaloblastic anemia Idiosyncratic neutropenia, thrombocytopenia
Antituberculous	Isoniazid	Tuberculosis and other mycobacteriae	Sideroblastic anemia (pyridoxine responsive), PRCA^a^, severe neutropenia
	Rifampicin		Thrombocytopenia in up to 10%
	Rifabutin		Neutropenia (mild to severe)
Antifungal	Amphotericin B	Antifungal	Anemia, leukopenia, severe thrombocytopenia
	Fluconazole		Thrombocytopenia
Chemotherapy	Various protocols and combinations	Malignancy	Anemia, neutropenia, thrombocytopenia, pancytopenia
Anticoagulant	Heparin (unfractionated or low molecular weight)	Thrombosis treatment or prophylaxis	Thrombocytopenia^b^

Sources: [22–24, 40–45].

Abbreviations: ART, antiretroviral therapy; Hb, hemoglobin; PRCA, pure red cell aplasia.

^a^Request polymerase chain reaction to exclude parvovirus B19. Consider bone marrow biopsy to confirm pure red cell aplasia.

^b^Consider 4 T's score for heparin-induced thrombocytopenia and thrombosis [45].

### Thrombocytopenia

Immune thrombocytopenic purpura (ITP) occurs in up to 30% of PWH and is mediated by an HIV-induced autoantibody generated against a platelet-surface glycoprotein with premature splenic platelet immune-mediated destruction. Patients may present with ITP as the first manifestation of HIV [46]. Evaluation of a peripheral blood smear is essential to exclude pseudothrombocytopenia from platelet clumping, which requires no treatment, and to exclude red cell fragments. Management of HIV-associated ITP includes ART initiation in newly diagnosed/noncompliant patients with ITP therapy in accordance with international guidelines [47]. A BM biopsy is unnecessary in most cases of ITP, but may be of value in refractory cases or where a splenectomy is being considered [48]. Therapeutic response rates for ITP in PWH are similar to patients with non-HIV-associated ITP, with about 70% requiring second-line therapy after 48 months of follow-up [49]. Poor response to first-line therapy generally mandates referral for specialized care. For patients with refractory HIV-associated ITP, splenectomy is safe and effective [50].

**Figure 3.**
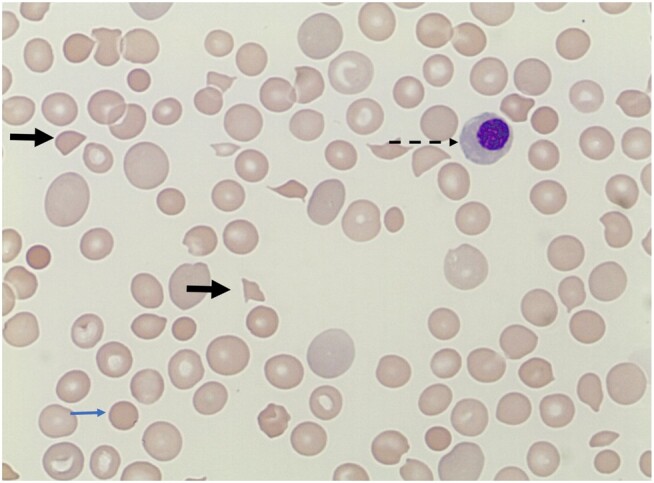
Human immunodeficiency virus–associated thrombotic thrombocytopenic purpura. The blood smear is essential for diagnosis, typically showing severe thrombocytopenia, marked red cell fragments (thick arrows), polychromasia (large immature bluish-tinged red cells), microspherocytes (thin arrow), and occasional nucleated red cells (dashed arrow).

Untreated HIV is a cause of acquired TTP initiated by platelet microthrombi in arterioles and capillaries [51]. Circulating red blood cells are mechanically sheared as they pass through microcirculatory microthrombi, leading to intravascular hemolysis and organ ischemia [52]. The hematological laboratory hallmarks are severe thrombocytopenia (typically <30 × 10^9^/L) combined with anemia and red cell fragmentation on the blood smear (Figure 3). The characteristic clinical pentad of TTP including severe thrombocytopenia, microangiopathic hemolytic anemia (MAHA), fluctuating neurological signs, fever, and renal impairment may not be present [53]. Thus, a high index of suspicion for TTP should be present in every PWH presenting with anemia and thrombocytopenia. Laboratory parameters including a coagulation screen, LDH, and haptoglobin levels may be helpful in excluding other causes of MAHA. Disseminated intravascular coagulation, which typically presents with extensively deranged coagulation parameters compared to TTP, should be routinely excluded [54]. LDH is highly elevated in TTP, and reducing levels predict clinical improvement. Most cases of HIV-associated TTP are caused by autoantibodies to an enzyme (ADAMTS13) that cleaves von Willebrand factor, and low plasma levels of these antibodies are diagnostic; however, testing may not be widely available in resource-constrained settings. Clinical risk calculators for TTP such as the PLASMIC score are widely available and well-validated, and help to delineate between TTP and other causes of MAHA [55].The standard of care for HIV-associated TTP is ART initiation, corticosteroids, and daily plasma replacement until resolution, either through plasmapheresis or with plasma infusions [56].

### Drug-Induced Cytopenias

Drug-induced cytopenias are common in PWH and may be caused by multiple drugs (Table 1). The 2023 national ART clinical guideline in SA includes isoniazid and cotrimoxazole for TB and *Pneumocystis jirovecii* pneumonia prophylaxis, respectively. Isoniazid can cause pyridoxine-responsive sideroblastic anemia, PRCA, and severe neutropenia [40, 57], whilst megaloblastic anemia may complicate cotrimoxazole therapy. The current recommended first-line ART regimen in SA for both adults and children from 10 years of age includes tenofovir, lamivudine, and dolutegravir, an integrase inhibitor [58]. Zidovudine causes hematological toxicity, which has limited its use in first-line ART regimens. Rifampicin, used in first-line TB therapy, may cause thrombocytopenia in up to 10% of recipients and, more rarely, leukopenia [41]. Intravenous antifungal therapy with amphotericin B may cause severe thrombocytopenia [42]. If a new cytopenia develops on ART, a drug-induced cytopenia should be considered and managed by dose adjustments [59].

## MALIGNANCIES

An adequate tissue biopsy sample is imperative for accurate histopathological diagnosis of malignancy. In TB-endemic areas such as SA, lymphadenopathy is usually due to TB or lymphoma, and more rarely other malignancies [60]. Histological diagnosis is essential to differentiate these conditions and can be accurately performed in an outpatient setting using a core biopsy handheld device, which expedites diagnosis [60]. In SA, a rapid-access outpatient lymph node core biopsy clinic has been successfully implemented using an online referral system. Medical doctors without specialist surgical or radiological training can competently perform these biopsies under local anesthetic with a handheld biopsy device [8, 60]. Fine needle aspiration (FNA) and cytology of a lymph node show low sensitivity for lymphoma or TB unless coupled with a cell block for lymphoma or molecular testing for TB [60]. Furthermore, inappropriate use of FNA cytology is linked to diagnostic delays and poorer patient outcomes [8, 60]. For disseminated aggressive B-cell NHL, BM aspirate samples can be submitted for morphology and flow cytometry analysis, where available, which rapidly assist diagnosis [61, 62]. HL is not readily diagnosed on BM aspirate nor body fluid samples due to a paucity of Hodgkin tumor cells. Lung cancer and other solid tumors, seen with increasing prevalence in PWH in the ART era, may present with lymph node and/or BM involvement causing cytopenias, A leukoerythroblastic blood smear picture (with red and white cell precursors seen) and teardrop red blood cells suggest metastatic BM involvement [6]. A BM biopsy should be performed for an unexplained leukoerythroblastic reaction on the blood smear after other causes, such as acute infection, have been excluded [63]. Recommendations for BM biopsy in PWH are provided in Table 2.

**Table 2.** Recommendations for Bone Marrow Biopsy in People With Human Immunodeficiency Virus

Indication	Recommendation(s)
General indications	Lymphadenopathy/mass unsuitable for biopsy CXR/CT scan suggests tuberculosis/lymphoma Unexplained cytopenias ± B symptoms^a^ Unexplained leukoerythroblastic blood smear ± teardrop red blood cells Blasts/lymphoma cells on blood smear
Bone marrow aspirate^b^	Routine stains of glass slides (including iron stain) Mycobacterial and fungal cultures: all lymphoma staging and possible lymphoma/leukemia Flow cytometry: 5 mL EDTA sample^c^: all cases of new/possible lymphoma/leukemia Cultures for cytogenetic karyotyping^d^: all new/possible cases of lymphoma/leukemia
Bone marrow trephine biopsy	Two unilateral posterior superior iliac spine cores with total length of >25 mm^e^ Special stains guided by clinical setting and bone marrow morphology

Abbreviations: CT, computed tomography; CXR, chest radiograph; EDTA, ethylenediaminetetraacetic acid.

^a^Exclude platelet clumping, red cell fragmentation, spherocytes, malaria, parvovirus B19, hematinic deficiencies, renal dysfunction, liver dysfunction.

^b^Stain trephine imprint slides where dry aspirate.

^c^Room temperature, to reach referral laboratory within 24 hours.

^d^Heparin tube or cytogenetic culture medium.

^e^To increase diagnostic yield for lymphoma [64].

### Aggressive B-Cell Lymphomas

Aggressive B-cell NHLs have an 11.5-fold increased risk in the ART era and are associated with Epstein-Barr virus (EBV) and HHV8 coinfection [6]. Postulated pathogenic mechanisms include chronic B-cell antigen stimulation and cytokine dysregulation. Patients often present late with advanced-stage, bulky disease [65]. ART has decreased mortality for lymphoma, and prognosis now approaches that seen in HIV-uninfected populations in well-resourced settings [66, 67]. However, outcomes are poor in sub-Saharan Africa because of late presentation and/or diagnosis [61, 68, 69]. PWH with aggressive lymphoma may develop superior mediastinal syndrome or spinal cord compression, which require urgent management within hours. In these cases, diagnostic biopsy should be expedited and steroid therapy ideally given after diagnosis, since steroids may obscure the pathological diagnosis [70].

Diffuse large B-cell lymphoma (DLBCL) is the most common lymphoid malignancy in adults worldwide and the most common HIV-associated lymphoma, followed by Burkitt lymphoma/leukemia (BL), HL, and plasmablastic lymphoma [64, 71]. Patterns of lymphoma in PWH have changed in the ART era, with a reduction in the incidence of DLBCL and primary central nervous system lymphoma; however, both BL and HL have remained stable or increased [67, 71]. Profound immunosuppression and prolonged viremia greatly increase the risk of HIV-associated DLBCL, which has various morphological variants and molecular subtypes [72]. Together with appropriate ART use and infection prophylaxis, similar chemotherapy regimens are used for HIV-associated DLBCL as in the general population. These include cyclophosphamide, doxorubicin, vincristine, and prednisolone (CHOP); rituximab (R)–CHOP; and, more recently, dose-adjusted etoposide, prednisone, vincristine, cyclophosphamide, and doxorubicin (DA-EPOCH). Treatment of DLBCL with R-CHOP is feasible and can achieve good outcomes in resource-restricted settings, with 74% 5-year overall survival reported from a single SA center in 73 HIV-negative patients [73]. A Ugandan study including PWH treated for NHL showed feasibility of CHOP and DA-EPOCH regimens with >50% 1-year survival [74]. In PWH with CD4^+^ T-cell counts ≥150 cells/µL and DLBCL in sub-Saharan Africa, overall survival approaches 50% at 3 years. However, outcomes are substantially worse for those with CD4^+^ T cell counts <150 cells/µL, with a median survival of 6 months [68].

BL is a highly aggressive tumor with very rapid doubling times. Disease may be localized to lymph nodes or tissues (Burkitt lymphoma) or disseminated in blood and BM (Burkitt leukemia). Characteristic laboratory features include translocations of the *MYC* gene leading to uncontrolled cellular proliferation. HIV-associated BL is frequently associated with leukemic presentation, complex cytogenetic karyotypes, and adverse clinical outcomes [61]. Any rapidly dividing tumor, including HIV-associated DLBCL and BL, may develop potentially lethal tumor lysis syndrome (TLS) [75]. TLS is characterized by hyperkalemia, hyperuricemia, hyperphosphatemia, hypocalcemia, and renal dysfunction and requires careful clinical management. BL in PWH responds well to intensive combination chemotherapy with or without rituximab [76], although outcomes are inferior in resource-constrained settings [77].

Plasmablastic lymphoma (PBL), an aggressive HIV-associated NHL originally described in the oral cavity, is common in SA and has not reduced in incidence in the ART era [64, 78]. Most PBL patients present at advanced stage with frequent BM involvement [78, 79]. PBL carries a poor prognosis with a median progression-free survival of 6 months; however, it does respond to chemotherapy and involved field radiotherapy, enabling many to achieve temporary remission [80, 81]. CHOP remains a commonly used chemotherapy regimen, particularly in resource-constrained settings [81]. Less common hematological malignancies occurring with increased frequency in PWH include primary effusion lymphoma and primary central nervous system lymphoma [65].

### Hodgkin Lymphoma

In PWH, HL is one of the commonest lymphomas [71, 82]. In well-resourced settings, HL is associated with moderate immunosuppression and CD4^+^ T-cell counts >200 cells/µL [6, 83]; however, in SA, HIV-associated HL frequently presents with advanced HIV and lower median CD4^+^ T-cell counts [84]. BM involvement at diagnosis is present in approximately 60% of HIV-associated HL cases in the SA setting. HIV-associated HL with BM involvement has an aggressive clinical course and poor survival rates, although outcomes in HIV-associated HL without BM infiltration match outcomes for HIV-uninfected HL [84]. HIV-associated HL is invariably associated with EBV-infected tumor cells compared with 20%–40% of non-HIV HL. EBV is considered oncogenic and immortalizes tumor cells, although the exact pathogenic role of EBV in HIV-associated HL remains unclear [85].

### Myeloma

Myeloma, a clonal plasma cell disease, is increased in prevalence in PWH in some studies, although this is not consistent. In sub-Saharan Africa, this may reflect underreporting and early death from other causes. HIV does not directly infect plasma cells, and the role of HIV in the pathogenesis of plasma cell disorders is unknown. Myeloma occurs at a younger age in PWH and commonly presents with end-organ disease (bone pain, anemia, and renal impairment) [86, 87]. Myeloma is associated with relatively high CD4^+^ T-cell counts in PWH and typically presents with an immunoglobulin G (IgG) paraprotein, implying a possible relationship between myeloma and an IgG response to HIV antigens [88]. Of note, reactive BM plasmacytosis is commonly found in PWH and should be distinguished from clonal disease.

### Other Malignancies

The incidence of non-AIDS-defining cancers has increased in the ART era, as the lifespan of PWH has improved [6]. Rare hematological malignancies such as peripheral T-cell lymphoma and natural killer cell lymphoma are also increased in PWH. Solid tumors with increased incidence include cervical cancer (AIDS-defining) and HIV-associated malignancies like lung cancer and hepatocellular cancer. These solid tumors may present with lymphadenopathy, cytopenias, and/or BM infiltration. PWH are also more likely to present with cancer at an advanced stage and to have poor outcomes with higher mortality [89].

## COAGULATION DISORDERS

As the efficacy of ART improves and PWH live longer, cardiovascular disease is increasing in prevalence in PWH [90]. PWH have a 2-fold increased prevalence of clonal hematopoiesis of indeterminate potential, the expansion of clonal hematopoietic stem cells due to leukemogenic mutations, which is associated with increased risks of coronary artery disease [91, 92]. PWH have significantly increased rates of myocardial infarction (risk ratio, 1.79 [95% confidence interval {CI}, 1.54–2.08]) and stroke (risk ratio, 2.56 [95% CI, 1.43–4.61]) and a 2- to 10-fold increased risk of VTE, which is associated with increased D-dimer levels [93]. Prothrombotic risk in HIV is linked to chronic inflammation with endothelial cell dysfunction, platelet activation, and coagulation factor activation and the production of antiphospholipid antibodies with a positive lupus anticoagulant [94]. There is increased release of von Willebrand factor by an activated endothelium and release of Factor VII and fibrinogen from the liver with concurrent downregulation of the natural anticoagulant proteins, Protein S and Protein C. Platelet activation and upregulation of tissue factor expression by circulating leukocytes may be initiated by HIV infection itself, opportunistic infections, or malignancy [90, 93, 95]. VTE risk is highest in PWH with advanced HIV disease, coexisting infections, and malignancies. Coagulation abnormalities improve with ART but do not normalize completely. Although lupus anticoagulants prolong clotting tests, in vivo they are associated with a thrombotic tendency and lupus anticoagulant positivity is associated with a significantly increased risk of thrombosis in PWH [94]. In PWH requiring anticoagulation, careful consideration of drug–drug interactions is critical as many ARTs interact with oral anticoagulants. The choice and duration of anticoagulation therapy and/or secondary prophylaxis is generally the same as for HIV-uninfected patients [96]. Rarely, acquired hemophilia with severe bleeding may occur in PWH, due to an acquired autoantibody to Factor VIII, which is reflected by a prolonged activated partial thromboplastin time test [97].

## CONCLUSIONS

Hematological complications in PWH are common and multifactorial. A thorough and systematic approach is essential, considering potential contributory factors such as medications and BM infiltration by opportunistic infections and/or malignancies. In cases of unexplained cytopenias, a BM biopsy proves to be a valuable diagnostic tool. HIV-associated lymphomas frequently manifest with advanced, aggressive disease. There is an increased incidence of both venous and arterial thrombosis, and a high index of suspicion needs to be maintained. The initiation of ART is a key element of treatment, with clinical management specifically tailored to each complication.

## Notes

***Author contributions.*** J. O. conceptualized the article content and wrote the initial manuscript. All authors reviewed, edited, and approved the final manuscript.

***Patient consent.*** This manuscript has met the ethical requirements of the Human Research Ethics Committee of the Faculty of Health Science, University of Cape Town.

***Financial support.*** E. V., J. O., and V. L. are supported by funding from the Fogarty International Center and the National Heart, Lung, and Blood Institute of the National Institutes of Health (award number 5D43TW010345-02).
